# The potential of astragalus polysaccharide for treating diabetes and its action mechanism

**DOI:** 10.3389/fphar.2024.1339406

**Published:** 2024-04-10

**Authors:** Shiyu Liu, Luyao Wang, Zehua Zhang, YuLin Leng, Yan Yang, Xiaoxu Fu, Hongyan Xie, Hong Gao, Chunguang Xie

**Affiliations:** ^1^ Hospital of Chengdu University of Traditional Chinese Medicine, Chengdu, Sichuan, China; ^2^ TCM Regulating Metabolic Diseases Key Laboratory of Sichuan Province, Chengdu, Sichuan, China; ^3^ Department of Endocrinology, Hospital of Chengdu University of Traditional Chinese Medicine, Chengdu, China

**Keywords:** astragalus polysaccharide, diabetes mellitus, insulin resistance, immunomodulation, diabetes complications

## Abstract

Type 2 diabetes presents a significant global health burden and is frequently linked to serious clinical complications, including diabetic cardiomyopathy, nephropathy, and retinopathy. Astragalus polysaccharide (APS), extracted from *Astragalus membranaceus*, exhibits various biochemical and physiological effects. In recent years, a growing number of researchers have investigated the role of APS in glucose control and the treatment of diabetes and its complications in various diabetes models, positioning APS as a promising candidate for diabetes therapy. This review surveys the literature on APS from several databases over the past 20 years, detailing its mechanisms of action in preventing and treating diabetes mellitus. The findings indicate that APS can address diabetes by enhancing insulin resistance, modulating the immune system, protecting islet cells, and improving the intestinal microbiota. APS demonstrates positive pharmacological value and clinical potential in managing diabetic complications, including diabetic retinopathy, nephropathy, cardiomyopathy, cognitive dysfunction, wound healing, and more. However, further research is necessary to explore APS’s bioavailability, optimal dosage, and additional clinical evidence.

## 1 Introduction

As of 2021, the global diabetic population reached 529 million, with diabetes standing as a leading cause of blindness, renal failure, heart attacks, stroke, and lower limb amputation (GBD, 2021 Diabetes Collaborators, 2023). While glucose-lowering therapies remain fundamental in diabetes management, the pursuit of novel therapeutics has gained momentum in research and development (Nauck et al., 2021). Herbal medicine emerges as a promising avenue, offering the potential for diversified diabetes treatment and improved patient quality of life (Tian et al., 2019; Ai et al., 2020; Zhang et al., 2022). Its efficacy stems from various mechanisms, including inhibition of α-glucosidase and α-amylase to reduce carbohydrate digestion and absorption, protection of pancreatic β-cells, enhancement of insulin sensitivity, promotion of gluconeogenesis and glycogen storage in the liver and muscle, antioxidant defense against organ damage, and attenuation of tissue inflammation to shield impaired tissues (Sun et al., 2021).

Polysaccharides are prominent constituents of herbal plants, and in recent decades, polysaccharides isolated from various herbs have exhibited a range of biological activities, including antitumor, antioxidant, hypoglycemic, antiradical, antiviral, hypolipidemic, and immunomodulatory effects. Polysaccharides from herbs such as *Astragalus membranaceus*, *Angelica sinensis*, *Cordyceps sinensis*, and *Ophiopogon japonicus* possess antidiabetic properties (Zeng et al., 2018).

A. membranaceus, initially documented in the Shennong Ben Cao Jing (Classic of the Divine Husbandman’s Materia Medica), refers to the dried root of the leguminous plants A. membranaceus Bge. var. mongholicus (Bge.) Hsiao and A. membranaceus (Fisch.) Bge (Chinese Pharmacopoeia Commission, 2020) and can be applied in the treatment of conditions such as weakness, wounds, anemia, fever, allergies, chronic fatigue, loss of appetite, uterine hemorrhage, and uterine prolapse (Kim et al., 2003). Studies have verified the immunomodulatory, antioxidant, anti-inflammatory, and anti-tumor properties of A. membranaceus, leading to its widespread use in various diseases like cardiovascular diseases (Su et al., 2021), diabetes mellitus (DM) (Tian et al., 2016), cancers (Sheik et al., 2021), respiratory diseases (Yang C.-G. et al., 2023), and neurological disorders (Xia et al., 2020).

Astragalus polysaccharides (APS) can be classified into heteropolysaccharides, glucans, neutral polysaccharides, and acid polysaccharides, with their monosaccharide composition varying based on the astragalus source and polysaccharide molecular weight (Tang and Huang, 2022). The complex chemical structures of individual APS make their isolation and characterization challenging, resulting in limited knowledge of APS composition. To date, 30 polysaccharides have been isolated and identified from *A. membranaceus* using extraction methods like water extraction, microwave extraction, enzyme extraction, and alkaline water extraction (Guo et al., 2018). Wang et al. utilized various techniques to analyze APS structure and composition, revealing rhamnose, galacturonic acid, glucose, galactose, and arabinose as components, with Glc as the primary constituent. Gas chromatography indicated eight main glycosidic bond types, with 1,4-glucose predominating, and NMR spectroscopy confirmed the α-configuration of isohead hydrogen (Wang et al., 2017). Yan et al. obtained APS by alcohol precipitation of astragalus extract followed by Sevage deproteinization, yielding three APS fractions (APS-1, APS-2, and APS-3) via DEAE-resin column chromatography (Yan et al., 2012).

Numerous studies have highlighted the potential of APS in treating Type 1 diabetes mellitus (T1DM), Type 2 diabetes mellitus (T2DM), and diabetic complications such as diabetic retinopathy (DR), diabetic nephropathy (DN), diabetic cardiovascular disease, and diabetic cognitive dysfunction, through various pathways (Wu et al., 2007; Dun et al., 2016; Sun S. et al., 2019; Meng et al., 2020; Ma, 2022). However, the broad scope of APS’s effects necessitates a detailed description to fully comprehend its role in diabetes. Therefore, this review summarizes APS’s role and underlying molecular mechanisms in diabetes and its related complications.

## 2 Methods

Studies related to APS’s anti-diabetic effects were identified through major scientific databases (PubMed, Web of Science, Embase, Google Scholar, and China National Knowledge Infrastructure) over the last 20 years. Some articles were also discovered through citation tracing or by visiting journal websites. Keywords used during the search included APS, astragalus membrane, astragalus montana, antidiabetic, hypoglycemic, hypolipidemic, mechanism, insulin sensitivity, and insulin resistance.

## 3 Effects of APS in DM

DM is a chronic disease characterized by hyperglycemia (Mathis et al., 2001). T1DM is a chronic, immune-mediated disease characterized by the destruction of insulin-producing β-cells in the pancreas (Norris et al., 2020), while T2DM is primarily characterized by insulin resistance and impaired insulin secretion (DeFronzo et al., 2015).

APS improves both T1DM and T2DM through different molecular mechanisms. In streptozotocin combined with a high-fat diet (HFD)-induced diabetic rats, APS (700 mg/kg, orally) for 8 weeks significantly reduced fasting plasma glucose, random blood glucose, glycated hemoglobin, and homeostatic model assessment of insulin tolerance (Zou et al., 2009). In another study in diabetic rats with streptozotocin/HFD, APS (400 mg/kg, orally) treatment for 5 weeks significantly reduced random blood glucose and improved insulin sensitivity in diabetic rats (Wu et al., 2005). In KKAy mice, APS improved hyperglycemia and systemic insulin sensitivity and reduced hepatic triglyceride and free fatty acid content (Mao et al., 2007). Furthermore, in Goto Kakizaki (GK) rats, APS (500 mg/kg, orally) for 8 weeks resulted in reduced body weight, area under the curve of postprandial blood glucose, and total cholesterol, triglycerides, and low-density lipoprotein cholesterol levels (Wei et al., 2018). In a study using APS (200 mg/kg, orally) to prevent the onset of diabetes in non-obese diabetes (NOD) mice, it was found that APS-administered NOD mice had a lower incidence of T1DM than the controls (Chen et al., 2008). The molecular mechanism of APS’s hypoglycemic effect involves its action on insulin-sensitive organs such as the liver, skeletal muscle, adipose tissue, and pancreas (Figure 2).

### 3.1 Improvement of insulin resistance by APS

Insulin resistance is characterized by an insulin-mediated defect in glucose metabolism control, particularly in the muscles, adipose tissues, and liver (Roden and Shulman, 2019; James et al., 2021). The insulin-sensitizing effect of APS has been extensively documented (Mao et al., 2009; Zhang et al., 2015; Sun J. et al., 2019). Adipose tissue is a major insulin target, and impaired glucose uptake in adipose tissue is linked to insulin resistance (Abel et al., 2001). The use of mouse 3T3-L1 preadipocytes is common in studying the insulin-sensitizing activity of hypoglycemic compounds. Zhang et al. (2018) used 3T3-L1 preadipocytes and APS (0.1 μg/mL) for intervention, demonstrating that APS increased preadipocyte proliferation in a dose-dependent manner, increased mRNA and protein content of glucose transporter protein 4, enhanced tyrosine phosphorylation of insulin receptor substrate 1 and phospho-Akt content, and increased AMP-activated protein kinase (AMPK) content. Ke et al. (2017) found that APS (1 μg/mL) suppressed miR-721 expression and increased PPAR-γ expression, promoting glucose uptake and enhancing insulin sensitivity in 3T3-L1 adipocytes in a dose- and time-dependent manner, through the miR-721-PPAR-γ-PI3K/AKT-GLUT4 signaling pathway.

The liver plays a central role in glucose synthesis and metabolism and is a major target organ for insulin resistance (Rodríguez-Gutiérrez et al., 2018). Wei et al. (2018) used a T2DM rat model established with GK rats and administered APS (500 mg/kg/day, orally) for 8 weeks. The results showed that APS attenuated insulin resistance in T2DM by upregulating or maintaining hepatic miR-203a-3p expression levels and by decreasing GRP78, CHOP, pJNK1, and cysteine asparaginase-12 protein expression levels. GU et al. (2015) administered APS (700 mg/kg, orally) for 8 weeks in GK rats and found that APS elevated hepatic PPARα, FGF21, and SIRT1 expression levels, reduced chronic inflammation, and partially attenuated hepatic steatosis, inhibiting aberrant glucose-lipid metabolism and insulin resistance.

**FIGURE 1:**
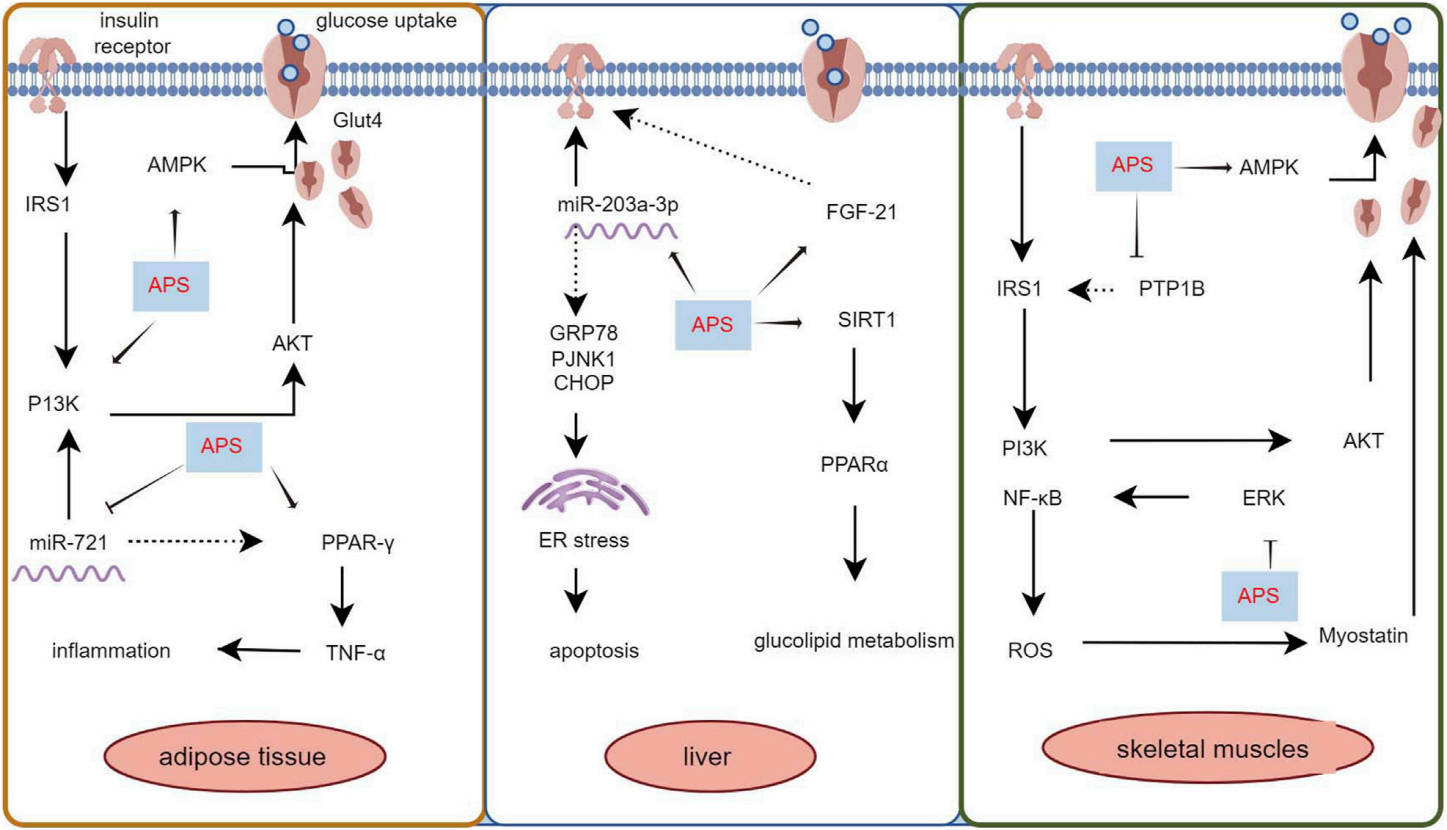
Mechanism of APS to improve insulin resistance in adipose tissue, liver and skeletal muscle.

Skeletal muscle, constituting 40%–50% of total body mass, is the primary tissue responsible for insulin-dependent glucose utilization (Sinacore and Gulve, 1993). Myostatin, a growth factor secreted by skeletal muscle, plays a pivotal role in regulating insulin signaling and insulin resistance (Guo et al., 2009). Liu M. et al. (2013) employed an HFD to induce diabetes in KKAy mice and treated them with APS (700 mg/kg, orally) for 8 weeks. The results revealed that APS treatment reduced myostatin expression and malondialdehyde production in skeletal muscle of non-insulin-dependent diabetic KKAy mice, ameliorated hyperglycemia, hyperlipidemia, and insulin resistance. *In vitro* studies using saturated acid palmitate-induced C2C12 cells showed that APS treatment (200 μg/mL) reduced reactive oxygen species (ROS) overproduction, extracellular signal-regulated kinase activation, and nuclear factor kappa B function, partially restored impaired glucose uptake, improved insulin sensitivity, and reduced myostatin expression in skeletal muscle. Glucose processing in skeletal muscle is regulated by the AMPK signaling pathway. Zou et al. (2009) intervened in HFD combined with streptozotocin-induced diabetic rats using APS (700 mg/kg, intragastric) and found that APS alleviated glucose toxicity by increasing hepatic glycogen synthesis and skeletal muscle glucose translocation through AMPK activation in T2DM rats. Cellular assays further demonstrated that APS treatment (400 μg/mL) significantly increased glucose uptake in L6 myotubes in a time- and concentration-dependent manner, promoted AMPK activation mediated by Ca2+/calmodulin-dependent protein kinase β or liver kinase B1 (Liu J. et al., 2013). Additionally, Liu et al. (2010) treated 12-week-old diabetic KKAy mice with APS (700 mg/kg) for 8 weeks and found that APS-treated diabetic mice showed partial restoration of insulin-induced phosphorylation of protein kinase B Ser-473 and glucose transporter protein 4 translocation in skeletal muscle. Wu et al. (2005) used APS (400 mg/kg, orally for 5 weeks) in HFD combined with streptozotocin-treated diabetic rats and observed that APS decreased the protein level and activity of protein tyrosine phosphatase 1B in the muscle of type 2 diabetic rats, increased insulin-induced tyrosine phosphorylation of insulin receptor β-subunits and insulin receptor substrate-1. Collectively, these studies demonstrate that APS can improve insulin resistance in adipose tissue, liver, and skeletal muscle through various pathways, highlighting its potential as an insulin sensitizer for type 2 diabetes treatment (Figure 1).

### 3.2 Improvement of pancreatic islet cell function by APS

Impaired insulin secretion by pancreatic β-cells is a core pathomechanism of T2DM, where disease progression hampers insulin secretion’s ability to maintain glucose homeostasis, leading to hyperglycemia (Weyer et al., 1999; DeFronzo et al., 2015). Pancreatic β-cells are crucial in maintaining glucose metabolism balance. APS has been shown to improve pancreatic β-cell number and function in diabetic rats through various protective mechanisms (Cui et al., 2016; Tang et al., 2017; Yang Z.-M. et al., 2021).

**FIGURE 2:**
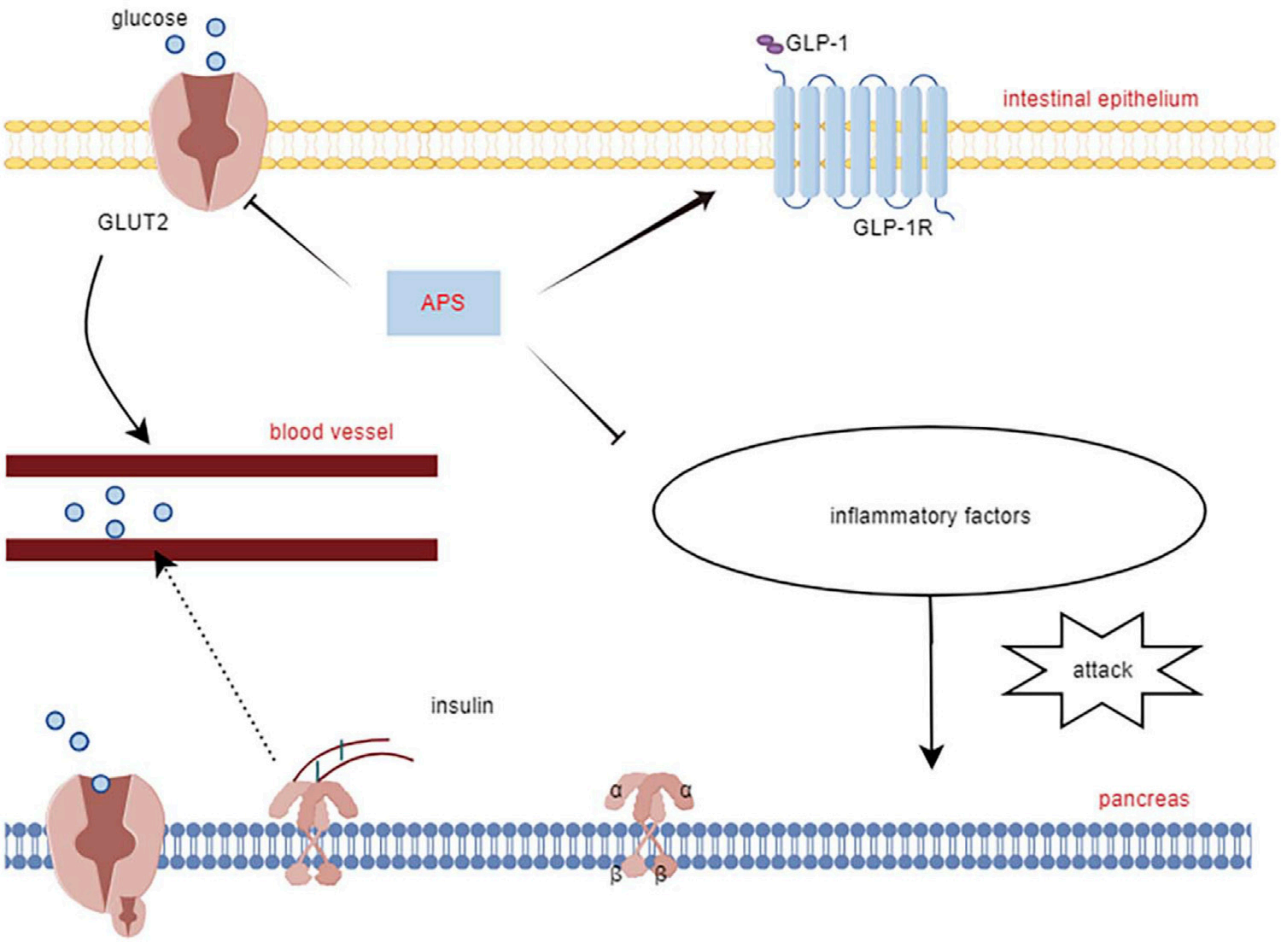
Mechanisms of APS to improve islet cell function.

Yang Z.-M. et al. (2021) reported that APS treatment reversed the decreased glucagon-like peptide-1 and its receptors expression levels in the pancreas of T2DM diabetic rats, along with increased expression of glucose transporter 2, promoting restoration of insulin secretion levels by affecting the STR/GLP-1/GLP-1R pathway in the enteropancreatic axis of T2DM rats. Deng et al. (2021) treated MIN6 cells (mouse pancreatic β-cell line) with APS (50, 100, and 200 μg/mL) after high glucose (HG) with palmitic acid treatment. APS-treated MIN6 cells exhibited higher viability, increased insulin secretion and pancreatic and duodenal homeobox 1 expression, and reduced apoptosis, reversing the effects of HG/palmitic acid on MIN6 cells. Additionally, chronic low-grade inflammation plays a crucial role in the development of metabolic disorders and diabetes mellitus. Excessive release of cytokines can severely damage pancreatic islet cells (Greenfield and Campbell, 2006). APS suppresses interleukin (IL)-1β protein production and the expression of several pro-inflammatory genes (e.g., iNOS, IL-1β, IL-6, MCP-1, and CD11c), which may also contribute to APS’s protective mechanism for islet cells (Lu et al., 2013). This underscores APS’s ability to safeguard pancreatic islets by reducing inflammatory factors, inhibiting islet β-cell apoptosis, and promoting insulin secretion (Figure 2).

### 3.3 Effects of APS in immunomodulation

APS has immunomodulatory effects on various immune cells, inhibiting over-activation of the immune system, and is widely used as an immunomodulator in clinical practice (Li et al., 2022). T1DM is an autoimmune disease mediated by T cells that destroy insulin-producing beta cells in the pancreatic islets (Marfil-Garza et al., 2021). It is associated with an imbalance between T helper 1 (Th1) and T helper 2 (Th2) subpopulations of helper T-lymphocytes and their cytokines. Th1 cytokines, such as interferon-gamma (IFN-γ), promote islet inflammation and DM, whereas Th2 cytokines, such as IL-4, protect pancreatic islet β-cells from damage (Roep, 2003).

**FIGURE 3:**
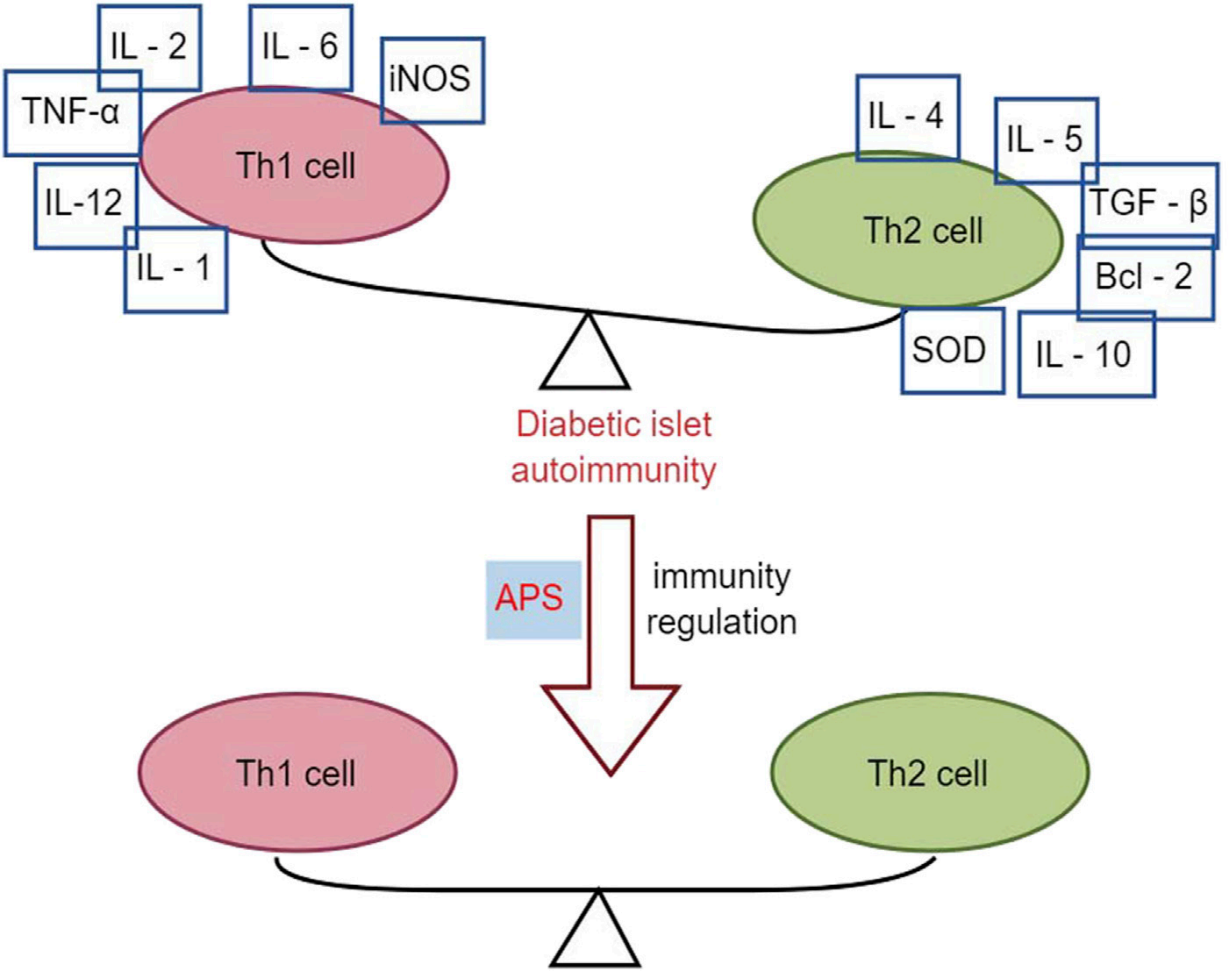
Effects of APS in immunomodulation.

Research has demonstrated that intervention with APS (2.0 mg/kg p. o.) in NOD mice for 10 weeks resulted in reduced infiltration of pancreatic islets with CD4^+^ T-lymphocytes, lower spleen T-lymphocyte CD4+/CD8+ ratios, and decreased gene expression of Th1-type cytokines in the pancreas compared to control NOD mice. This led to a decrease in the conversion of Th1-type cytokines to Th2 cytokines, including IL-4, IL-5, IL-10, transforming growth factor (TGF), B-cell lymphoma-2 (Bcl-2), and superoxide dismutase (SOD), thereby altering the autoimmune response and delaying or preventing the development of T1DM in NOD mice (Chen et al., 2008). Furthermore, another study using APS (2 g/kg p. o.) to intervene in NOD mice for 2 months found that early application of APS pre-immunization significantly downregulated the expression levels of Fas and iNOS genes in the pancreatic islets of NOD mice. Simultaneously, it upregulated the expression levels of Bcl-2 and SOD genes in the pancreatic islets, correcting the immune imbalance of oxidative or apoptotic death in NOD mice (Chen et al., 2007). In multiple low-dose streptozotocin (MLD-streptozotocin)-induced diabetic mice treated with APS (100, 200, and 400 mg/kg i. p.) for 15 or 30 days, serum insulin concentration was upregulated, the β-cell mass increased, the percentage of apoptotic β-cells decreased, the Th1/Th2 cytokine ratio was downregulated, and peroxisome proliferator-activated receptor γ gene expression was upregulated, suggesting that APS could act through immunomodulation of the Th1/Th2 cytokine ratio (Li et al., 2007). Zhou et al. (2011) demonstrated that APS (100 mg/kg, 400 mg/kg p. o.) intervention in streptozotocin-induced diabetic mice for 15 days reduced pancreatic islet β-cell damage by modulating galactoglucan-1 (gal-1) and down-regulating the Th1 response, leading to apoptosis of CD8 T cells. Overall, APS increased total β-cell mass in T1DM mice by reducing apoptosis of pancreatic β-cells and protecting regenerating β-cells from damage through immunomodulation (Figure 3).

### 3.4 Effects of APS in gut microorganisms

The gut microbiota plays a significant role in metabolism and immune regulation, acting as an endocrine organ. Dysbiosis of the gut microbiota and disruption of the intestinal barrier can lead to organ damage in patients with diabetes (Yang G. et al., 2021).

Chen et al. (2022) demonstrated that in HFD combined with streptozotocin-induced diabetic mice, APS intervention (400 mg/kg/day, orally) for 6 weeks strongly inhibited the potential pathogen *Shigella* and promoted the growth of beneficial bacteria such as *Eubacterium rectale* and *Lactobacillus species*. Additionally, APS repaired intestinal microbiota, remodeled specific intestinal barrier damage, reduced lipopolysaccharide and systemic inflammation, and improved metabolic parameters in T2DM mice (Chen et al., 2022). Yang B. et al. (2023) treated streptozotocin-induced type 1 diabetes mice with APS-1 (200 mg/kg, orally) for 8 weeks, showing that APS-1 modulated the expression of zona occludens 1, occludin, and claudin-1, improving intestinal barrier function. APS-1 also restored the relative abundance of *Trichoderma reesei*, *Lactobacillus reesei*, and *Faecalibaculum*, inhibiting inflammatory responses, protecting pancreatic islet cells, reducing blood glucose, and improving insulin resistance by increasing the relative abundance of intestinal flora (Yang B. et al., 2023). The effects of APS on gut microbes were further demonstrated in db/db mice, where APS treatment (600 mg/kg, orally) for 16 days increased the production of fecal short-chain fatty acids. This improvement in short-chain fatty acids production enhanced the expression of G-protein-coupled receptors 41/43 and tight junction proteins (occudin and zona occludens 1) restored the diabetic community, improved gut integrity, and alleviated diabetes symptoms in db/db mice (Song et al., 2022).

**FIGURE 4:**
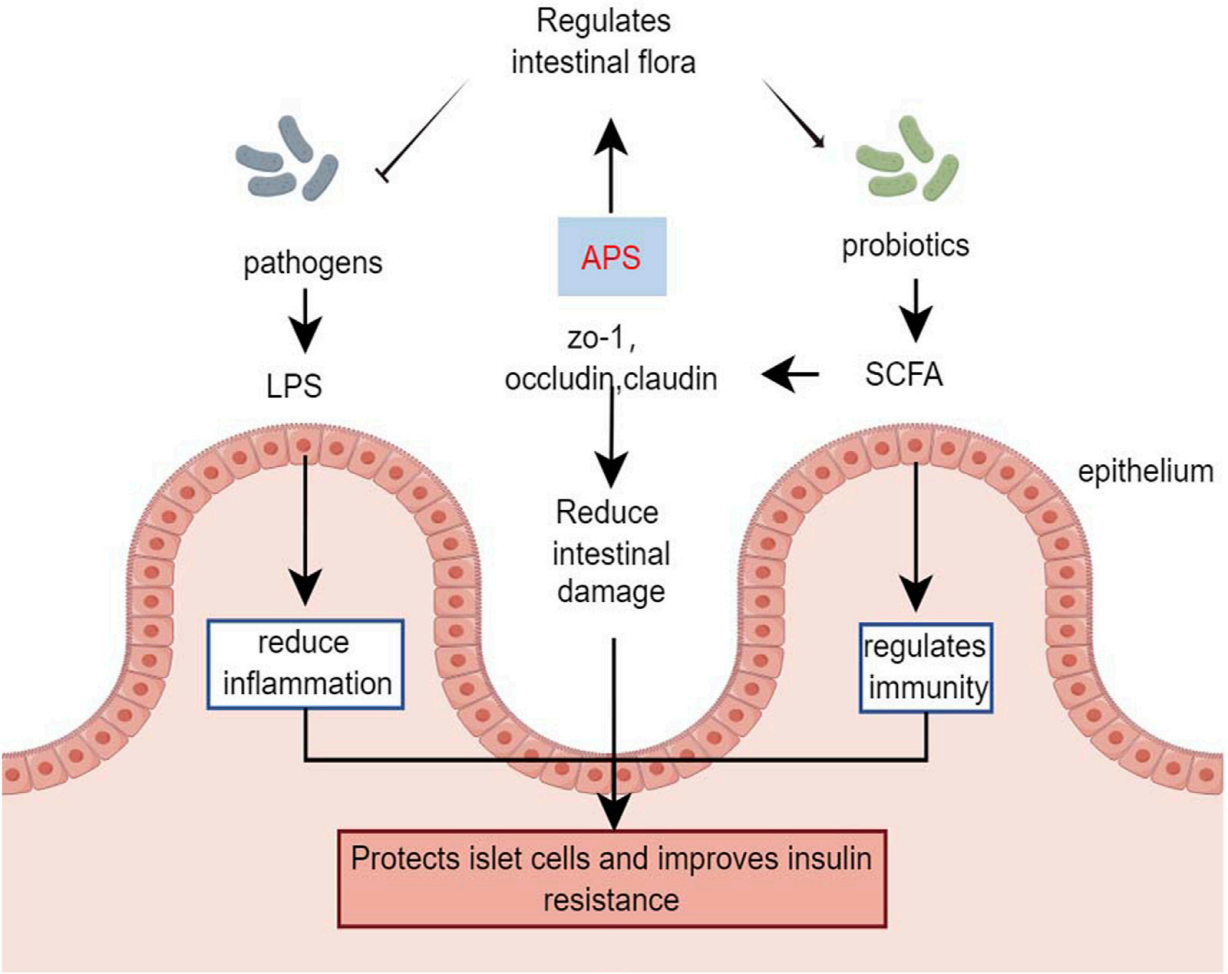
Effects of APS in gut microorganisms.

Butyrate-producing bacteria are crucial for human health, providing energy to the intestinal epithelium, maintaining intestinal bacterial balance, and regulating host cellular responses (Louis et al., 2010). However, a clinical study involving *in vitro* fermentation of fresh feces from healthy donors and patients with T2DM found that after 48 h of fermentation with APS, the organic acid profile of APS fermentation was more influenced by individual differences in gut microbiota than in the healthy and T2DM groups (Xu et al., 2023). Animal studies have indicated that APS improves the profile of intestinal flora and restores the intestinal barrier in diabetic mice. However, the role of APS in regulating the intestinal flora of individuals with diabetes remains controversial and requires further research (Figure 4).

## 4 Effects of APS on diabetes complications

**FIGURE 5:**
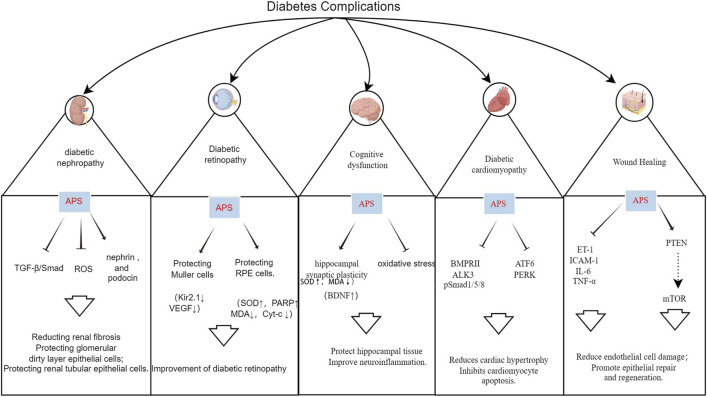
Mechanism of action of APS in the treatment of multiple diabetic complications.

Hyperglycemia is a primary cause of diabetes-related morbidity and mortality, leading to various vascular complications. Oxidative stress and inflammation are key factors in these complications. APS has shown promise in ameliorating these complications through various pathways (Figure 5; Supplementary Table S1).

### 4.1 APS and DN

DN is characterized by persistent albuminuria and progressive decline in renal function, affecting up to 50% of patients with diabetes, and is a leading cause of end-stage renal disease (ESRD), associated with increased cardiovascular morbidity and mortality (Selby and Taal, 2020). APS has a protective effect on DN, delaying its development through podocyte repair, reduction of tumor necrosis factor, inhibition of renal tubular epithelial cell apoptosis, and improvement of oxidative stress (Li et al., 2011; 2018; Guo et al., 2018; Meng et al., 2020).

TGF-β1, a key member of the TGF-β family, plays a central role in the TGF-β/Smad signaling pathway, promoting fibrosis. Its overexpression stimulates the growth of renal fibroblasts, increases extracellular matrix content, and leads to renal sclerosis. Controlling TGF-β1 expression is beneficial for ameliorating diabetic-induced renal damage, making it a significant target in the treatment of DN (Voelker et al., 2017). APS attenuates renal lesions by down-regulating TGF-β1 protein content and mRNA overexpression in the kidneys of diabetic rats (Lai et al., 2002). Li et al. (2018) demonstrated that APS (200 and 400 mg/kg, orally) inhibited the renal TGF-β1/Smads signaling pathway in diabetic rats after 8 weeks of intervention, significantly reducing fasting glucose, blood creatinine, and urea nitrogen. Meng et al. (2020) also showed that different doses of APS (25, 50, and 100 mg/kg, orally) reduced kidney weight, 24-h urinary microalbumin, blood urea nitrogen, creatinine, collagens III and IV, transforming growth factor-β3, α smooth muscle actin, and Smad3 levels in HFD combined with streptozotocin-induced diabetic rats after 8 weeks of intervention. APS protects the kidney from interstitial fibrosis by suppressing the TGF-β/Smad signaling pathway and reducing extracellular matrix formation.

Epithelial cell, or podocyte, injury in the glomerular basement membrane is a critical factor in the formation of DN proteinuria (Barutta et al., 2022). Li et al. (2011) administered APS (400 mg/kg/day, orally) to streptozotocin-induced diabetic rats for 8 weeks. The results showed significant reductions in blood glucose, blood urea nitrogen, blood creatinine, and total 24-h urinary protein. Renal pathological changes were attenuated, and the expression of the major podocyte-specific proteins nephrin and podocin was increased in the rats with APS intervention, suggesting that APS’s preventive effect on DN may be related to the maintenance of podocyte integrity (Li et al., 2011).

Early in the development of DN, renal tubular cells undergo apoptosis and oxidative damage. Apoptosis of renal tubular epithelial cells further promotes interstitial fibrosis and atrophy, while the overproduction of ROS is a primary initiator of diabetic complications and a key factor in cellular damage (De Nicola et al., 2014). Guo et al. (2018) demonstrated that human renal tubular epithelial cells (HK-2) treated with APS showed increased survival, decreased apoptosis, and reduced ROS content. This indicates that APS could promote HG-induced proliferation and inhibit apoptosis and transdifferentiation of HK-2 cells (Guo et al., 2018).

In clinical use, APS has been developed as APS injection and APS fFlush, with clinical studies confirming that APS flush can treat DN, with lipid-lowering effects and a reduction in the urinary albumin excretion rate (Cao and Zhang, 2007). APS injection can reduce early activated T-lymphocytes, improve cellular immune function, and enhance the body’s immune level in older patients with DN after 3 weeks of treatment (Deng et al., 2014). However, further research via controlled, large-scale clinical trials is still required.

### 4.2 APS and DR

DR is a significant ocular complication of diabetes and is the leading cause of blindness and visual impairment in individuals with diabetes worldwide. The prevalence of DR is expected to increase annually (Teo et al., 2021; Tan and Wong, 2023). Li et al. (2008) demonstrated that administering APS (700 mg/kg p. o.) to streptozotocin combined with HFD-induced diabetic rats for 8 weeks led to a decrease in the expression of Kir2.1 protein in retinal Muller cells at an early stage, thereby protecting the Muller cells and reducing the incidence of DR. Ke et al. (2010) cultured second-generation glial fibrillary acid protein-positive Müller cells for 3 days using 400 μg/mL APS and 20 mmol/L glucose in a normal medium, resulting in a significant reduction in the expression of vascular endothelial growth factor in Müller cells in the high-glucose APS group. This suggests that APS prevents and treats DR by reducing the expression of vascular endothelial growth factor in Müller cells (Ke et al., 2010). Additionally, a study demonstrated that administering APS solution (700 mg/kg p. o.) for 8 weeks to high-fat-fed KKAy mice reduced blood glucose levels, enhanced insulin sensitivity, and attenuated the expression of TNF-α, thereby ameliorating retinopathy in diabetic KKAy mice (Wu et al., 2007).

The retinal pigment epithelium is a crucial target for DR initiation after hyperglycemia, and microRNAs can inhibit the expression of target genes by directly targeting the 3′ untranslated regions of genes at the post-transcriptional level (Shukla et al., 2011). Cellular experiments demonstrated that APS could increase cleaved-ATF6; Bax; p-PERK; p-IRE-1; Bcl-2; cleaved caspase-12, -9, -3; and unleaved SOD and PARP levels; it could also decrease malondialdehyde and mitochondrial Cyt-c levels, ameliorate HG-induced oxidative stress, mitochondrial damage, endoplasmic reticulum stress and apoptosis, and alleviate the metabolic memory of HG-treated retinal pigment epithelium cells. These findings suggest that APS has a potential therapeutic role in the development of DR (Liu et al., 2019; Peng et al., 2020).

### 4.3 APS and diabetic cardiomyopathy (DCM)

Cardiovascular events are the leading cause of death in patients, and DCM is a pathological condition caused by DM. DCM is initially characterized by myocardial fibrosis and associated diastolic dysfunction, followed by the manifestation of systolic dysfunction and ultimately clinical heart failure (HF) (Jia et al., 2018). DCM results from the interaction of several factors, including hyperinsulinemia, hyperglycemia, oxidative stress, abnormal fatty acid metabolism, and cardiac autonomic neuropathy (Acar et al., 2011; Dillmann, 2019).

Cardiac hypertrophy is a major feature of DCM, with HG inducing cardiac hypertrophy, and multiple signaling pathways involved in this process. Bone morphogenetic protein 10, a cardiac peptide growth factor, plays a specific role in cardiac hypertrophy and is considered an influential target for its treatment (Sun et al., 2023). Sun et al. (2023) demonstrated that APS had a potent anti-hypertrophic effect on HG-stimulated H9c2 cardiomyocytes and streptozotocin-induced DCM rats. In animal experiments, APS (0.5, 1, 2 g/kg p. o.) administered to streptozotocin-induced diabetic rats for 16 weeks reduced the expression of BMPRII, ALK3, and p-Smad1/5/8, alleviated cardiac hypertrophy, and improved cardiac function by inhibiting the activation of the bone morphogenetic protein 10 pathway in a dose-dependent manner (Sun et al., 2023).

Apoptosis is considered an important contributor to DCM, leading to cardiac cell loss, reduced cardiac contractility, and ultimately cardiac remodeling (Cai et al., 2006). Sun et al. showed that *in vivo*, APS (1 g/kg p. o.) intervention in streptozotocin-induced T1DM rats for 16 weeks downregulated the protein expression of activating transcription factor 6 and protein kinase RNA-like ER kinase. *In vitro*, APS inhibited HG-induced apoptosis in H9C2 cells and reduced the expression of ATF6 and PERK-related proteins in the endoplasmic reticulum stress pathway. These findings confirmed that APS enhanced cardiac function and alleviated myocardial apoptosis in diabetic conditions through *ex vivo* and *in vivo* studies (Sun et al., 2017; Sun et al., 2019 S.). In conclusion, APS can protect the myocardium via its anti-hypertrophic and anti-apoptotic effects on cardiomyocytes.

### 4.4 APS and cognitive dysfunction

Cognitive impairment and dementia, including Alzheimer’s disease, are increasingly recognized as common complications and comorbidities of both T1DM and T2DM, with individuals with diabetes having a 2.4–1.25 times higher risk of cognitive impairment compared to the general population (Biessels et al., 2020). Mechanistic studies provide various pathophysiological clues, including dysmetabolic disorders, brain insulin resistance, vascular endothelial dysfunction, accumulation of glycosylation end products, neurodegeneration, and inflammation (Ehtewish et al., 2022). Several studies have demonstrated that APS can significantly reduce the latency period of locomotion navigation experiments, decrease the dwell time of space exploration experiments, and improve performance in water maze experiments in diabetic rats (Dun et al., 2016; Li et al., 2017; Yang, 2017). Yang (2017) administered APS (60 mg/kg p. o.) to rats fed a high-fat and high-sugar diet and observed potential improvement in hippocampal synaptic plasticity through increased expression of brain-derived neurotrophic factor in the hippocampus, thus enhancing learning and memory functions. In interventions by Dun et al. (2016); Li et al. (2017) in streptozotocin-induced Wistar rats for 8 weeks using varying doses of APS (200, 400, and 800 mg/kg p. o.), APS was found to enhance glucose-lipid metabolism, insulin resistance, and antioxidant capacity in diabetic rats. Moreover, APS elevated hippocampal tissue SOD activity and reduced malondialdehyde content, suggesting potential protection against diabetic-induced brain damage via anti-oxidative stress and anti-apoptotic effects on hippocampal tissue (Dun et al., 2016; Li et al., 2017).

Alzheimer’s disease is considered a metabolic disorder, with metabolic disturbances contributing directly to Alzheimer’s disease through various pathways, including synaptic disconnection, neuronal loss, accumulation of amyloid-β, and hyperphosphorylation of tau protein (de la Monte, 2014). Epidemiological data strongly suggest an association between type 2 diabetes and an increased risk of dementia (Hamzé et al., 2022). After administration of APS (700 mg/kg p. o.) for 4 weeks to intervene in HFD combined with streptozotocin-injected APP/PS1 double transgenic mice, Huang et al. (2017) observed reduced insulin resistance and hepatic triglyceride levels induced by metabolic stress, decreased astrocytosis and microglial activation in the vicinity of plaques, and alleviation of metabolic stress-induced diabetes and subsequent neuroinflammation, thereby improving behavior in these transgenic mice.

### 4.5 APS and wound healing in diabetic

Diabetic foot ulcers are a significant concern, affecting 15% of individuals with diabetes and posing a serious threat to their quality of life, often leading to lower limb amputations (Okonkwo and DiPietro, 2017; Yue et al., 2022; McDermott et al., 2023). The complex pathogenesis of these ulcers involves inflammation, angiogenesis, and extracellular matrix remodeling, which impede proper wound healing in patients with diabetes (Chang and Nguyen, 2021). The literature highlights the crucial role of APS in managing diabetic wound healing.

Phosphatase and tensin homolog (PTEN) are variably expressed in diabetic patients, and their downregulation can delay wound healing in those with diabetic foot ulcers (Xu et al., 2020). Ma’s study used APS (50, 100, or 200 mg/kg p. o.) in streptozotocin-induced diabetic rats with skin wounds. The results demonstrated that APS reduced endothelial damage by inhibiting the release of inflammatory mediators such as ET-1, ICAM-1, IL-6, and TNF-alpha levels. Moreover, APS upregulated PTEN and suppressed the mTOR pathway activation, facilitating wound healing in diabetic rats (Ma, 2022). Additionally, the combination of APS with different drug carriers for novel wound management materials is a promising avenue of research. In a diabetic rat model, APS-loaded tissue-engineered scaffolds enhanced periwound cutaneous blood flow, increased endocrine expression, and boosted microvessel density in regenerating skin tissues, leading to improved wound healing in a dose-dependent manner (Yang et al., 2015; Ma, 2022). Another *in vivo* animal experiment reported that nanofibrous membranes loaded with APS and astragaloside IV curbed wound inflammation, promoted collagen fiber deposition, regenerative epithelial repair, and significantly accelerated wound healing in diabetic rats (Yue et al., 2022). These studies underscore the potential of APS as a therapeutic agent in promoting diabetic wound healing. However, clinical trials investigating the efficacy of APS in treating diabetic foot ulcers are currently lacking.

## 5 Conclusion and directions

DM and its complications are major chronic non-communicable diseases that significantly impact quality of life. While current clinical treatments can effectively control symptoms and slow disease progression, they often fail to prevent multi-organ damage and functional failure. Therefore, developing new effective treatments for diabetes is crucial for improving patients’ quality of life, leading to a focus on novel molecular drugs targeting diabetic complications.

APS, a natural plant extract, shows promise in hypoglycemia and treating diabetic complications. *In vitro* studies and animal models have demonstrated APS’s effectiveness in treating DM and its complications, including DR, DN, DCM, diabetic cognitive dysfunction, and diabetic wound healing. However, these studies often focus on initial assessments of pharmacodynamic effects, necessitating further investigation into dose-effect relationships and toxic side effects. While studies indicate that polysaccharides from Chinese herbal medicine, like APS, have significant hypoglycemic activity without toxic side effects or adverse reactions, there is a lack of research specifically on APS’s toxic side effects and adverse reactions. Therefore, studies on its safety and toxicity are necessary before considering it for human studies. The complex chemical structure of polysaccharides, including APS, limits our understanding of their exact composition and structure, hindering clinical studies and resulting in a lack of high-quality clinical trials. Thus, future research should focus on conducting more clinical studies based on thorough structural characterization, along with pharmacokinetic and pharmacotoxicity studies. Furthermore, exploring the exact mechanism, potential molecular targets, pharmacokinetics, pharmacodynamics, and side effects of APS on diabetes mellitus and its complications through clinical trials is crucial.
