# Supplementary material for: The potential of astragalus polysaccharide for treating diabetes and its action mechanism
Source: Front Pharmacol. 2024 Apr 10;15:1339406. doi: 10.3389/fphar.2024.1339406 (PMC11039829; doi:10.3389/fphar.2024.1339406)
Supplement: Supplementary file 1 [file Table1.DOCX]

Supplementary Material

**Supplementary Table 1**  Posological regimen of APS applied experimentally for the treatment of major diabetic complication

| Type of experiment | Model Methods | model | Dose/Dosing method/period | Diabetic complication | Beneficial effects and involved mechanisms | Title |
| --- | --- | --- | --- | --- | --- | --- |
| Animal experiment | STZ 55 mg/kg/day for 3 days, i.p Wistar rats | Type 1 diabetes | APS 200,400 mg/kg per day orally for 8 week | diabetic nephropathy | APS has a renoprotective effect on DM rats, and the mechanism may be related to the inhibition of the renal TGFβ1/Smad signalling pathway in DM rats. | (Li et al., 2018) |
| Animal experiment | feeding the rats a high-fat diet and injecting Single dose of STZ 35 mg/kg, i.p SD rats | Type 2 diabetes | APS 25, 50, and 100 mg/kg per day orally for 8 week | diabetic nephropathy | Astragalus polysaccharide significantly improved blood glucose and protected kidney function in a rat diabetes model. | (Meng et al., 2020) |
| Animal experiment | injecting Single dose of STZ 60 mg/kg, i.p SD rats | Type 1 diabetes | APS 400 mg/kg per day orally for 8 week | diabetic nephropathy | The protective effect of APS on the DN kidney may be related to the maintenance of podocyte nephrin and podocin expression. | (Li et al., 2011) |
| Cell experiment | HK-2 cells with 5.5/30 mmol/L glucose | / | APS 200 μg / L per day | diabetic nephropathy | APS promotes high glucose-induced proliferation and inhibits apoptosis and transdifferentiation of renal tubular epithelial cells by a mechanism related to the down-regulation of the JAK/STAT signaling pathway. | (Guo et al., 2018) |
| clinical trial | Elderly patients with early diabetic nephropathy | diabetes | APS injection 250 mg per day for 3 weeks | diabetic nephropathy | Astragalus polysaccharide injection has the effect of regulating immune function and reducing inflammation in elderly patients with early diabetic nephropathy, and can improve renal function. | (Deng et al., 2014) |
| Animal experiment | feeding the rats a high-fat diet and injecting Single dose of STZ 25 mg/kg, i.p SD rats | Type 2 diabetes | APS 700 mg/kg per day orally for 8 week | diabetic retinopathy | APS reverses the decrease in Kir2.1 protein expression in early retinal Muller cells of diabetic rats, thereby reducing the incidence of diabetic retinopathy. | (Li et al., 2008) |
| Cell experiment | Müller cells with 20 mmol/L glucose | / | APS 400 μg / L per day | diabetic retinopathy | APS prevents and treats diabetic retinopathy by reducing VEGF expression in MÜLLER cells. | (Ke et al., 2010) |
| Animal experiment | KKAy rats fed a high-fat diet | Type 2 diabetes | APS 800 mg/kg per day orally for 8 week | diabetic retinopathy | APS ameliorates retinopathy in diabetic KKAy mice by a mechanism related to attenuating TNF-α expression and improving insulin resistance. | (Wu et al., 2007) |
| Cell experiment | Human RPE cell line (ARPE-19) and rat primary RPE (PRPE) | / | APS at different doses | diabetic retinopathy | APS suppressed high glucose-induced metabolic memory in retinal pigment epithelial cells through inhibiting mitochondrial dysfunction-induced apoptosis by regulating miR-195. | (Liu et al., 2019) |
| Cell experiment | Human RPE cell line (ARPE-19) and rat primary RPE (PRPE) | / | APS 12.5,25,and 50μg/ ml per day | diabetic retinopathy | APS inhibited ER stress and subsequent apoptosis via regulating miR-204/SIRT1 axis in metabolic memory model of RPE cells. | (Peng et al., 2020) |
| Animal experiment | injecting Single dose of STZ 60 mg/kg, i.p SD rats | Type 2 diabetes | APS 50, 100, and 200 mg/kg per day orally for12 week | diabetic cardiomyopathy | APS can alleviate cardiac hypertrophy and prevent DCM by inhibiting activation of the BMP10 pathway.APS is a promising candidate for the treatment of DCM. | (Sun et al., 2023) |
| Animal experiment | a single injection of STZ 60 mg/kg i.p SD rats | Type 1 diabetes | APS 1g/kg per day orally for16 week | diabetic cardiomyopathy | APS could improve heart function and attenuate myocardial apoptosis in DCM rat model and down-regulate the protein expressions of activating transcription factor 6 (ATF6) and protein kinase RNA-like ER kinase (PERK) related factors of ER stress pathway. | (Sun et al., 2019b) |
| Cell experiment | H9C2 cells with 5.5/33 mM glucose | / | 0.8 mg/ml APS | diabetic cardiomyopathy | H9C2 cell apoptosis and the expressions of ATF6 and PERK related proteins of ER stress pathway. | (Sun et al., 2019b) |
| Animal experiment | SD rats were fed a high-fat, high-sugar diet for 8 weeks. | Rats on a high-fat, high-sugar diet | APS 500 mg/kg per day orally for 8 week | Cognitive dysfunction | APS improves learning memory and synaptic plasticity induced by high-fat and high-sugar diet in rats, and its mechanism is related to the increase of hippocampal BDNF expression. | (Yang, 2017) |
| Animal experiment | intraperitoneally injected once with 10% STZ (50 mg/kg) i.p Wistar rats | Type 1 diabetes | APS 200 ,400,800mg/kg per day orally for 8 week | Cognitive dysfunction | APS is an important active ingredient for improving memory in diabetic rats. Its potential mechanism of action is related to the effects of APS on glycolipid metabolism, antioxidant and IR, and it is a potential therapeutic candidate for the treatment of diabetic memory deficits. | (Dun et al., 2016) |
| Animal experiment | intraperitoneally injected once with 10% STZ (50 mg/kg) i.p Wistar rats | Type 1 diabetes | APS 200 ,400,800mg/kg per day orally for 8 week | Cognitive dysfunction | APS enhanced SOD activity in hippocampal tissue significantly and reduced MDA content, suggesting that APS may protect against diabetes-induced brain injury through anti-oxidative stress and anti-apoptotic effects. | (Li et al., 2017) |
| Animal experiment | Feeding a high-fat diet and injecting a single dose of STZ 50 mg/kg i.p | ameliorate metabolic stress-induced diabesity | APS 500mg/kg per day orally for 7 week | Cognitive dysfunction | Astragalus membranaceus-polysaccharides may be used to ameliorate metabolic stress-induced diabesity and the subsequent neuroinflammation, which improved the behavior performance in metabolically stressed transgenic mice | (Huang et al., 2017) |
| Animal experiment | injecting Single dose of STZ 45 mg/kg, i.p SD rats | Type 1 diabetes | APS 50 ,100,200mg/kg per day orally for 2 week | Diabetes mellitus combined with trauma | APS could promote wound healing in diabetic rats by upregulating PTEN and suppressing the mTOR pathway activation | (Ma, 2022) |
